# Pysim-sv: a package for simulating structural variation data with GC-biases

**DOI:** 10.1186/s12859-017-1464-8

**Published:** 2017-03-14

**Authors:** Yuchao Xia, Yun Liu, Minghua Deng, Ruibin Xi

**Affiliations:** School of Mathematics Science and Center for Statistical Science, Peking University, Yiheyuan Road 5, Beijing, 100871 China

**Keywords:** Copy number variation, Translocation, Breakpoints, Next-generation sequencing

## Abstract

**Background:**

Structural variations (SVs) are wide-spread in human genomes and may have important implications in disease-related and evolutionary studies. High-throughput sequencing (HTS) has become a major platform for SV detection and simulation serves as a powerful and cost-effective approach for benchmarking SV detection algorithms. Accurate performance assessment by simulation requires the simulator capable of generating simulation data with all important features of real data, such GC biases in HTS data and various complexities in tumor data. However, no available package has systematically addressed all issues in data simulation for SV benchmarking.

**Results:**

Pysim-sv is a package for simulating HTS data to evaluate performance of SV detection algorithms. Pysim-sv can introduce a wide spectrum of germline and somatic genomic variations. The package contains functionalities to simulate tumor data with aneuploidy and heterogeneous subclones, which is very useful in assessing algorithm performance in tumor studies. Furthermore, Pysim-sv can introduce GC-bias, the most important and prevalent bias in HTS data, in the simulated HTS data.

**Conclusions:**

Pysim-sv provides an unbiased toolkit for evaluating HTS-based SV detection algorithms.

## Background

Structural variations (SVs) are genomic variations that lead to structure changes of a donor genome. Indels, copy number variations (CNV) and genomic rearrangements are all subclasses of SVs. Many researches revealed that SVs are wide-spread in normal human populations [1, 2] as well as in cancer genomes [3–5]. High-throughput sequencing (HTS) has become a major platform for SV detection and a number of algorithms have been developed for SV detection with HTS data [6]. In genome studies based on HTS data, an important problem is to benchmark performances of various algorithms in different scenarios. The performance of an algorithm depends on its design, its implementation as well as quality and features of the sequencing data. An ideal method for benchmarking is by sequencing and subsequent experimental validation, but this method is expensive, labor intensive and time-costing. Thus, simulation of HTS data becomes a powerful and cost-effective alternative way for benchmarking genomic variation detection algorithms.

Usually, benchmarking HTS-based SV detection algorithms by simulation involves (1) generation of genomes containing simulated SVs and (2) simulation of HTS short reads based on the genomes with simulated SVs. To approximate real HTS data, the generated genomes should be similar to real genomes and the simulated HTS data should contain various sequencing errors and biases. Since tumor samples often contain normal contaminations and heterogeneous subclonal tumor cells, simulation data of tumor genomes should contain normal contamination and/or multiple subclonses.

There are several available SV simulation packages including RSVsim [7], SCNVsim [8], VarSim [9], IntSIM [10] and SInC [11]. These packages provide great resources for the community. However, as far as we know, no available package has systematically addressed all issues in data simulation for SV benchmarking. For example, RSVsim can simulate a wide range of SVs, but it cannot generate CNVs and cannot generate tumor data with normal contamination and subclones. SCNVsim considered issues in simulating tumor data such as aneuploidy, normal contamination and multiple-subclones, but it can only generate tumor genomes with somatic SVs/CNVs but not other types of somatic events such as single nucleotide variations (SNVs). VarSim can simulate comprehensive classes of genomic variations, but it also cannot generate tumor data with aneuploidy, normal contamination and multiple-subclones. IntSim and SInC are able to simulate both germline and somatic variants, but they can only simulate SNVs and CNVs. In addition, it is well-known that GC-bias is wide-spread in HTS data. Although the read simulator pIRS [12] can introduce GC-bias in the simulation data, its GC-bias profile was trained on one set of data and users can essentially only generate one type of GC-bias. Our analysis of hundreds of sequencing data from The Cancer Genome Atlas and the 1000 Genome project data revealed that GC-bias can take many different forms [13], and pIRS are not flexible enough to simulate these GC-biases.

Here, we present Pysim-sv for SV simulation. Compared with other HTS data simulation packages, Pysim has three main advantages:

1. It can simulate a full spectrum of SVs as well as SNVs;
2. It allows simulation of tumor data with aneuploidy, normal contamination and multiple subclones;
3. It can generate HTS data with GC-biases of any form.

## Methods

**Fig. 1:**
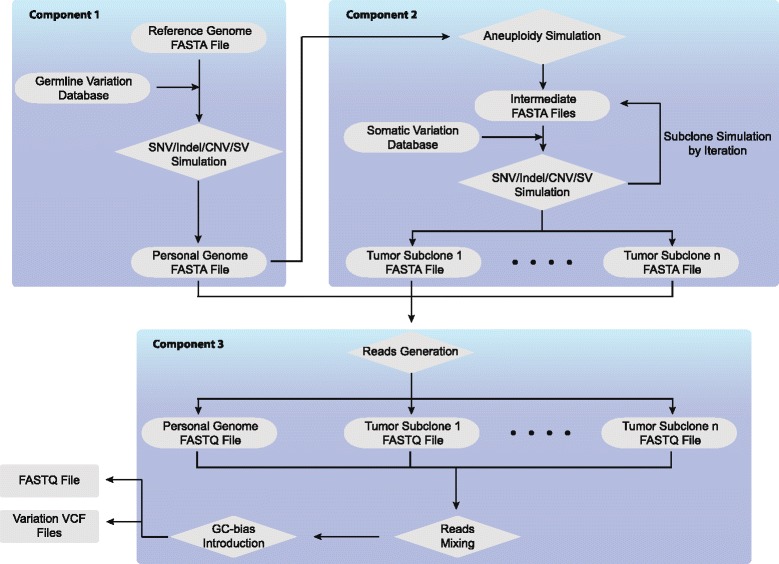
The workflow of Pysim-sv. Component 1 simulates a personal genome by introducing genomic variations to a given reference genome. Component 2 generates tumor genomes by simulating aneuploidy and somatic variations. Subclones are iteratively generated. Component 3 generates HTS reads, mixes reads from different tumor/normal genomes and introduces GC-bias

Pysim-sv uses fasta format reference genome as input. This tool consists of three major components (Fig. 1). The first component generates a personal genome by introducing germline SNVs, indels and SVs. The second component is for tumor genome simulation. If a user requires generating tumor data, Pysim-sv first simulates aneuploidy based on the personal genome simulated in the first component. Somatic variations and subclones are then simulated. The third component generates HTS reads and introduces GC-biases.

### SNV/indel simulation

Germline SNVs and indels are sampled from existing database such as dbSNP [14] or a VCF file provided by users. Somatic SNVs and indels are randomly placed in the genome, or if a tumor mutation database is given (such as COSMIC [15, 16]), they are randomly sampled from the given database. The SNVs can be heterozygous or homozygous. Users can control the parameters such as the number of SNVs/indels to be introduced and the heterozygous/homozygous ratio according to their simulation purpose.

### SV simulation

Pysim-sv can simulate seven classes of SVs including deletions, insertions, tandem duplications, inversions, intra-chromosomal translocations, inter-chromosomal translocations and CNVs. A deletion is generated by removing segments from the genome. An insertion is placed by inserting a sequence into the genome. The inserted sequence can come from either a database of known human insertion sequence (e.g. the Venter genome insertion sequence) or a series of random nucleotides with a random length. An inversion is generated by replacing a segment by its reverse complement. We simulate translocations by taking segments from the genome and inserting them to the same chromosomes (intra-chromosomal translocation) or different chromosomes (inter-chromosomal translocation). Translocations can be balanced (no gain/loss of genome segments) or unbalanced (gain/loss of genome segments). Hence, the original segments are either removed or kept in the original location, respectively. A copy number loss event is generated by removing a segment, and a copy number gain event is generated by randomly inserting a segment to several locations of the same chromosome.

Non-allelic homologous recombination (NAHR) and non-homologous recombination (NHR) are two major mechanisms of generating SVs [17]. Pysim-sv simulates NAHR by placing breakpoints in repeat regions in the RepeatMasker database [18] and simulates NHR by randomly placing breakpoints in the reference genome. As breakpoints often co -occurs with SNVs/indels [19], Pysim-sv also introduces SNVs/indels near SV breakpoints. Users can set the expected number of SNVs/indels near SV breakpoints. To make sure the reported SV breakpoints and SNV/indel positions are correct, the positions of SVs and SNVs/indels are first simulated and the SVs and SNVs/indels ordered according to their chromosome positions. Then, they are generated backwardly from the last position to the first first position.

### Tumor aneuploidy simulation

Aneuploidy is the deviation of ploidy number from the normal ploidy number. It is very common in cancer genomes and is related with chromosomal instability [20]. Aneuploidy has important impact on tumor CNV detection since it often causes incorrect estimation of copy numbers. Pysim-sv allows generation of aneuploidy according to user-specified aneuploidy status (the copy number of each chromosome) of the simulated genome. Pysim-sv generates genomes with aneuploidy from normal diploid genomes and the resulting genomes provide the starting genomes for somatic SV simulation.

### Tumor genome heterogeneity and purity simulation

Tumor cell populations often contain many heterogeneous subclones. Pysim-sv simulates new subclones from a progenitor genome by randomly placing new somatic variations. The number of new subclones from the progenitor genome can be specified by users. Iterative application of this procedure can be used to simulate the clone evolution model [3] as well as the cancer stem cell model [21]. After a specified number of subclones are simulated, Pysim-sv then simulates HTS short reads from these tumor/normal genomes, and mixes these reads according to user specified proportions of these genomes. By default, ART [22] is used to generate HTS data.

### GC bias introduction

**Fig. 2:**
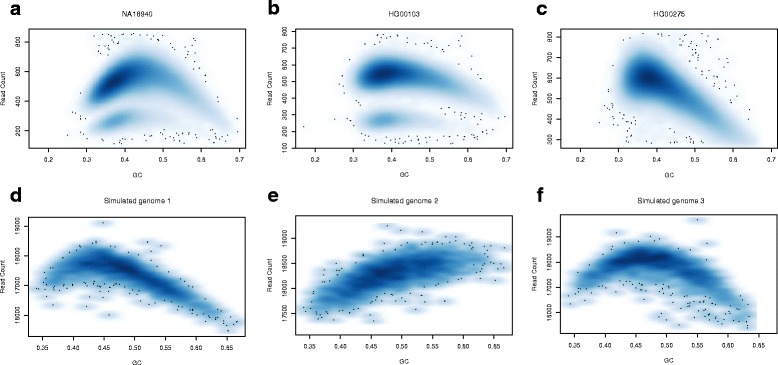
The GC-dependency in real data and simulated data. The GC-dependency in (**a**, **b**, **c**) three real sequencing data from the 1000 Genome Project and in (**d**, **e**, **f**) three simulated data generated by pysim-sv. The x-axis is the GC-proportion in 10 Kb bins and the y-axis is the number of mapped reads in the bins. Note that the lower bands in the *left* and *middle panel* of (**a**, **b**, **c**) correspond to bins in chromosome X and the two individuals here are two males. The functions in (**d**, **e**, **f**) are *f*_1_, *f*_2_ and *f*_3_ as presented in the GC-bias introduction section

After the initial HTS data are generated, we further employ a biased subsampling method to introduce GC-biases. Specifically, Pysim-sv first calculates a subsampling probability for every read pair (or every read for single-end data). This subsampling probability depends on the local GC-content of the sequence from which the read pair is generated. We use the following procedure to introduce GC-biases. Given a read pair (*R*_1_,*R*_2_), let (*S*_1_,*S*_2_) (*S*_1_<*S*_2_) be the positions in the simulated chromosome from which the read pair was sequenced. Note that these reads are Illunima platform type reads. Then, the sequence *S* from *S*_1_ to *S*_2_+*r* in the simulated chromosome is the segment that generates the read pair (r is the read length). Let *G* *C*_*S*_ be the GC proportion of this segment. Then, we will subsample this read pair (*R*_1_,*R*_2_) with probability *p*_*S*_=*f*(*G* *C*_*S*_)/(1+*f*(*G* *C*_*S*_)), where *f* is a user specified function. Specifically, Pysim-sv first generates a random number from the Bernoulli distribution with the success rate as the probability *p*_*S*_. Pysim-sv will or will not select this read pair depending on whether or not the random number is 1. Figure 2 show examples of GC-dependency in real sequencing data (top panel) and in simulated data by Pysim-sv (bottom panel). The GC-dependency functions in Fig. 2 d–f are chosen as *f*_1_(*x*)=−8.89*x* ^3^−3.56*x* ^2^+9.13*x*−1.58, *f*_2_(*x*)=−2.5(*x*−0.6)^2^+1, and

$$\begin{array}{@{}rcl@{}} f_{3}(x) = \left\{\begin{array}{ccc}-2x+1.8 & x~\geq 0.4\\ 4x-0.6 & 0.2\leq x\leq 0.4\\ 0.1 & \text{otherwise}. \end{array}\right. \end{array} $$

Note that the GC-dependency functions of Fig. 2 d–f are not chosen to be corresponding to GC-dependency of Fig. 2 a–c. Pysim-sv is very flexible in generating GC-bias and users can easily specify any GC-dependency functions.

## Results and Discussions

Based on the human reference genome (hg19), we simulated six genomes with three levels of purity (1, 0.5, and 0.8) and two levels of GC-dependency (with and without GC-bias). Each genome contains 100,000 SNVs and 200 SVs. We generated 30 × 100 bp paired-end read data. We used GATK [23] and Varscan [24] to detect SNVs in these simulated data, and used Delly [25], BreakDancer [6], GASV-Pro [26] and Meerkat [5] to detect SVs. As a comparison, we also ran these algorithms on a real data set NA12878 from the 1000 Genome Project. The NA12878 data was sequenced on the Illumina platform with a read length of 100 bp. The mean insert size is 320 bp with a standard deviation of 60 bp. The coverage of this data set is around 40 ×.

Table 1 shows the sensitivity and false discovery rate (FDR) of GATK and Varscan on the six simulated data. We found that Varscan had a higher sensitivity and FDR rate than GATK. The sensitivities of GATK and Varscan tend to decrease and their FDR rates tend to increase when the purity decreases. Similarly, compared with no GC-bias data, their sensitivities are lower and their FDRs are higher when GC-bias is introduced. On the NA12878 data, by comparing with reported SNVs of this individual from the 1000 Genome Project, the sensitivities of GATK and Varscan were 96.8 and 96.3%, similar to their performances on the simulated data set with purity 1.

**Table 1:** The sensitivity and false discovery rate (FDR) of the different SNV detection algorithms with different simulation setups

Purity	Subclone1	Subclone2	GC-bias	Methods	Sensitivity	FDR
Purity =1	1	0	Yes	GATK	0.97679	1.13×10^−4^
				Varscan	0.97892	2.36×10^−3^
			No	GATK	0.97826	9.20×10^−5^
				Varscan	0.98134	2.05×10^−3^
Purity =0.8	0.4	0.4	Yes	GATK	0.90253	1.93×10^−3^
				Varscan	0.92501	5.36×10^−3^
			No	GATK	0.90853	8.32×10^−4^
				Varscan	0.93001	5.05×10^−3^
Purity =0.5	0.4	0.1	Yes	GATK	0.81253	1.09×10^−2^
				Varscan	0.84501	5.34×10^−2^
			No	GATK	0.81853	2.11×10^−3^
				Varscan	0.85001	4.51×10^−2^

Note that the purity column represents the proportion of “tumor” cells in the simulated data. Subcolone1 and Subcolone2 columns represent the proportions of subcolone 1 and 2 in the simulated data, respectively. When purity is 1 and subclone1 is 1, it means that all data are from subclone1 and it essentially like a sequencing data from a normal genome

**Fig. 3:**
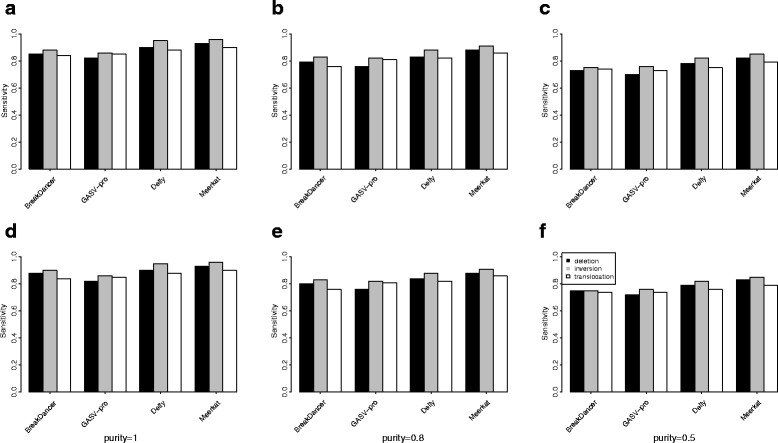
The sensitivity of the four SV detection algorithms with different parameters. Deletions (*black*), inversion (*grey*) and translocations (*white*) are compared, individually. **a**, **b**, and **c** are the simulated data with GC-bias, and **d**, **e**, **f** are the simulated data without GC-bias. The purities are 1 (**a**, **d**), 0.8 (**b**, **e**) and 0.5 (**c**, **f**)

**Fig. 4:**
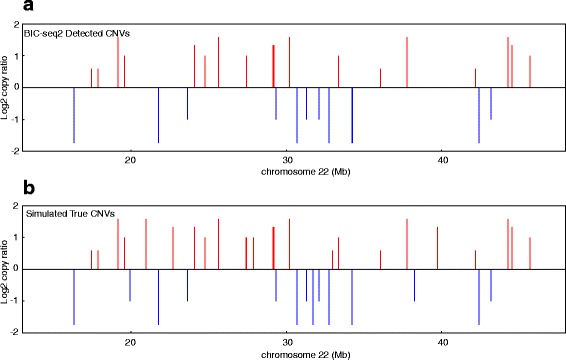
True CNVs in a simulated genome and detected by BIC-seq2. **a** Forty CNVs were introduced in the simulated genome and thirty-five copy number gains (*red lines*) and copy number losses (*blue lines*) were detected by BIC-Seq2. **b** True copy number gains (*red lines*) and copy number losses (*blue lines*) in the simulated genome

The sensitivities of the SV detection algorithms are shown in Fig. 3. Compared with the SNV detection algorithms, the sensitivities of these algorithms are relatively low. All methods have sensitivities above 75%. Meerkat and Delly achieved higher sensitivity rates than BreakDancer and GASV-Pro because Meerkat and Delly used both discordant reads and split reads to detect SVs. As the purity decreases, we also observe that the sensitivities of the SV detection algorithms tend to decrease. For NA12878, we compared the SV calls from the four SV detection algorithms with the golden standard SV set as reported in Mills et al. 2011 [1]. Since most of the reported SVs in Mills et al. 2011 are deletions, we only considered deletion predictions. The precision and recall rates of the four algorithms are calculated by comparing with the golden standard deletions (Table 2). The precisions of the four algorithms are relatively low, but this low level of precision might be due to the possibility that many true deletions are not included in the golden standard set. For CNV detection, we generated another simulation data containing 40 CNVs with their sizes ranging from 100 bp to 10 kb and their copy numbers ranging from 0 to 6. SNVs and Indels were also introduced to this genome by randomly sampling from the dbSNP database. We used Pysim-sv coupled with ART to generate 10 × data of 100 bp paired-end reads with GC-bias. BIC-seq2 [13, 27] was used to detect CNVs based on BWA [28] mapping. Among 40 CNVs, 35 were detected by BIC-Seq2 (Fig. 4).

**Table 2:** Overlaps of deletion predictions of the four SV detection algorithms with golden standard deletions in Mills el. al. 2011

Software	Total reported deletion	Deletions in golden standard	Precision	Recall rate
Delly	1075	358/545	0.33	0.66
BreakDancer	779	328/545	0.42	0.60
Gasv-pro	1382	339/545	0.25	0.62
Meerkat	687	372/545	0.54	0.68

To test Pysim-sv speed, we used a diploid human genome(hg19) as reference and evaluated the computational efficiency with different parameter settings. We simulated 2.4 million SNVs which were randomly selected in dbSNP. We simulated 1–3 subclones with 100–300 SVs ranging from 1 to 10 kb. The test was performed on a 32-core sever with Intel Xeon 2.40 GHz CPU, running a Linux operating system. The running time and the memory usage of Pysim-sv were summarized in Table 3.

**Table 3:** The running time (hour) and memory usage (Gb) for Pysim-sv simulations with different parameter settings

Simulation set up		Time	Memory
Genome Simulation^a^	One subclone with 100 SVs	0.98 h	9.1 Gb
	One subclone with 200 SVs	1.34 h	9.9 Gb
	One subclone with 300 SVs	1.73 h	11.6 Gb
	Two subclones with 100 SVs	1.03 h	9.5 Gb
	Three subclones with 100 SVs	1.28 h	10.2 Gb
Read generation^b^	Mixing reads from 2 subclones and 1 normal genome (392 M reads)	2.76 h	6.2 Gb
	GC-bias Introduction (130 M reads)	4.10 h	2.5 GB

^a^Time and memory usage for simulating subclone genomes and a normal genome

^b^Time and memory usage for read generation by ART are not shown

## Conclusion

In this paper, we present Pysim-sv to simulate HTS data for benchmarking SV detection algorithms. Pysim-sv can simulate a wide spectrum of germline and somatic variations and thus the simulated genomes are more similar to real genomes. Pysim-sv is the first HTS data simulation tool that can introduce the GC-bias in the simulated HTS data. These features make simulation data generated by Pysim-sv more similar to real HTS data. We believe that Pysim-sv is a useful toolkit for performance evaluation of SV detection and SNV detection algorithms based on HTS data.

## Availability and requirements

**Project name:** Pysim-sv**Project home page:** https://github.com/xyc0813/pysim/ **Operating system(s):** Windows,Unix-like (Linux, Mac OSX)**Programming language:** python(>=2.7)**Any restrictions to use by non-academics:** None

## Abbreviations

CNV: Copy number variation
HTS: High throughput sequencing
NAHR: Non-allelic homologous recombination
NHR: Non-homologous recombination
SVs: Structural variations
SNVs: Single nucleotide variations
